# Comparing learning retention in medical students using mixed-reality to supplement dissection: a preliminary study

**DOI:** 10.5116/ijme.6250.0af8

**Published:** 2022-04-29

**Authors:** Guy Baratz, Preethy S. Sridharan, Valeda Yong, Curtis Tatsuoka, Mark A. Griswold, Susanne Wish-Baratz

**Affiliations:** 1 Sagol School of Neuroscience, Tel-Aviv University, Tel-Aviv, Israel; 2 Department of Neurosciences, School of Medicine, Case Western Reserve University, Cleveland, OH, USA; 3 Department of Surgery, Temple University Hospital, Philadelphia, PA, USA; 4 Department of Population and Quantitative Health Sciences, School of Medicine, Case Western Reserve University School of Medicine, Cleveland OH, USA; 5 Department of Radiology, Interactive Commons, Case Western Reserve University, Cleveland, OH, USA; 6 Department of Anatomy, HoloAnatomy, School of Medicine, Case Western Reserve University, Cleveland, OH, USA

**Keywords:** Mixed-Reality, medical education, anatomy education, knowledge retention

## Abstract

**Objectives:**

To evaluate student impressions of learning anatomy with mixed-reality and compare long-term information retention of female breast anatomy between students who learned with a mixed-reality supplement and their classmates who dissected cadavers.

**Methods:**

In Part 1, 38 first-year medical student volunteers, randomly divided into two groups, completed a mixed-reality module and cadaveric dissection on the female breast in a counterbalanced design. Participants also completed post-quizzes and surveys. Part 2 was a non-randomized controlled trial, 8-months after completing Part 1 and 6-months after a final exam on this content. The performance of twenty-two Part 1 participants and 129 of their classmates, who only dissected, was compared on a delayed post-quiz. Wilcoxon signed-rank test, Mann-Whitney U test, and 95% confidence intervals were used to analyze the data.

**Results:**

In Part 1, the Wilcoxon signed-rank test determined that participants expressed significantly more positive responses to mixed-reality and found mixed-reality easier for learning and teamwork. In Part 2, the Mann-Whitney U test found mixed-reality participants scored significantly higher on a delayed-post quiz than their classmates who only dissected (U = 928, p < .009).

**Conclusions:**

This study suggests that medical students may prefer mixed-reality and that it may be an effective modality for learning breast anatomy while facilitating teamwork. Results also suggest that supplementing cadaveric dissection with mixed-reality may improve long-term retention for at least one anatomical topic. It is recommended that similar studies evaluate a larger sample and additional anatomical regions to determine the generalizability of these findings.

## Introduction

In recent decades, the rapid increase in computer-based technologies has changed the educational media with which learners can interact.^1^^, ^^2^ As such, like other academic institutions, medical schools might benefit from finding innovative ways to educate a new generation of learners raised in a rapidly evolving, technology-forward world, and who desire this to be reflected in their curricula for effective and efficient learning.^1^^-^^3^ Available technological advances have yet to be fully integrated into medical anatomy labs in a manner that effectively addresses the challenges of medical education today.^4^ Many medical schools across the United States continue to rely on cadaveric dissection or prosection to teach medical students fundamental anatomical concepts.^5^ These traditional modalities expose learners to anatomical variation, pathological conditions, accurate spatial representations of structures, and anatomical complexity.^6^^, ^^7^ Dissection also exposes students to death in a controlled setting and provides an opportunity, directly or indirectly, to confront their feelings and thoughts concerning death.^8^^-^^10^ Most medical professionals agree that cadaveric dissection is critical to attaining adequate knowledge of human anatomy and developing practical skills.^11^^-^^14^

Cadaveric dissection, however, has several limitations. Some consider the act of dissection to be an inefficient use of valuable curricular time, and medical schools across the United States have responded by reducing the total number of hours spent on gross anatomy.^13^^,^^15^^,^^16^  In some parts of the world, medical schools are opting to exclude cadavers from their curricula entirely.^8^ Additionally, cadaver labs are costly to maintain and require the use of hazardous chemical preservatives.^17^^-^^19^ The act of dissection is also challenging for a novice medical student, which can lead to errors that damage tissues or incomplete procedures that prevent students from learning the desired material. Even when dissection is performed correctly, many complex and critically important anatomical structures are too small or difficult to view.^20^^, ^^21^ Students must often rely on static, two-dimensional (2D) representations of these structures in textbooks or other references to supplement their dissections. It can be challenging to translate this information into an understanding of how structures are oriented and mutually related in three dimensions (3D).^4^ Regardless of whether dissection is employed to learn anatomy, anatomical "knowledge retention [is] an issue of concern" among medical students and educators.^22^

According to Custers, "the value of education depends largely upon the life span of what has been learned."^23^ A number of studies have supported the claim that over time there is a decline in retention of anatomy and other basic science knowledge.^22^^-^^25^ Technological advancements such as computer-assisted learning modalities have been incorporated into basic science laboratories, such as anatomy and physiology, to bolster learning.^2^

Mixed-reality (MR) is a form of technology that enables students to learn a given topic while interacting with peers and instructors through their headsets. Remote learning with MR has been shown to be effective in the operating room, enhancing and enabling logistical flexibility in medical anatomy curricula.^26^^-^^29^ Previous trials comparing cadaver-based dissection to MR showed students acquired the same knowledge at an accelerated time scale.^30^^, ^^31^ In addition, MR can be used to model structures that cannot be readily viewed in a cadaver, potentially extending the educational capacity of the device beyond that of the traditional dissection lab.^26^  Given the known advantages and disadvantages of cadaveric dissection, perhaps the sweet spot for medical education is a combination of both cadaveric dissection and MR.

This study explores the value of MR as an educational supplement by testing medical student satisfaction and long-term ability to retain anatomical knowledge when MR is used in addition to cadaveric dissection. To address this, an MR module was designed to supplement a cadaveric dissection lesson covering female breast anatomy. The female breast was selected for this study because its substructures are generally small and poorly visualized on a cadaver. Yet, knowledge of breast anatomy is fundamental for understanding the clinical progression of breast cancer and other pathologies.^32^

Medical education is replete with attempts to implement technologies that facilitate learning and make it more individualized and collaborative.^1^ However, according to Pickering, "there remains a paucity in empirical evidence detailing the quantifiable impact on learning gain…for individual learners…specifically between two points in time." ^33^

The purpose of the present study was to gather student feedback about their experience using MR as a supplement to cadaveric dissection and to compare long-term retention of information about female breast anatomy between students who supplemented their initial learning with MR and their classmates who did not.  We set the following hypotheses:

    ·   Students would react more positively to MR than to dissection and find understanding and learning the required material easier with MR than dissection.

    ·   Students would find MR to be easier to perform than dissection and easier to work with as a team.

    ·   Students who supplemented cadaveric dissection with MR would perform better on their final exam than their peers who only dissected.

    ·   Students who supplemented cadaveric dissection with MR would perform at least as well as their peers who only dissected on a delayed post-test.

## Methods

### Study design and participants

A two-part study was conducted to assess student satisfaction and knowledge retention, comparing students who learned using MR and students who did not. Part 1 consisted of a cross-over study in which volunteers were randomly divided into two groups. Each group completed both an MR module and cadaveric dissection on the female breast during the thoracic anatomy portion of a gross anatomy course. Forty-three first-year medical students from Case Western Reserve University School of Medicine (CWRU SOM) initially volunteered to participate in Part 1 of this study. At the time of the intervention, six students chose not to participate, leaving a total of thirty-seven volunteers. The order in which groups completed the modules in Part 1 was counterbalanced, with a randomly distributed group of volunteers completing the MR module first, and the other group dissecting first. Six weeks after Part 1, the entire class completed a final exam, including material from the breast module. Part 2 took place six months after the final exam.

**Figure 1:**
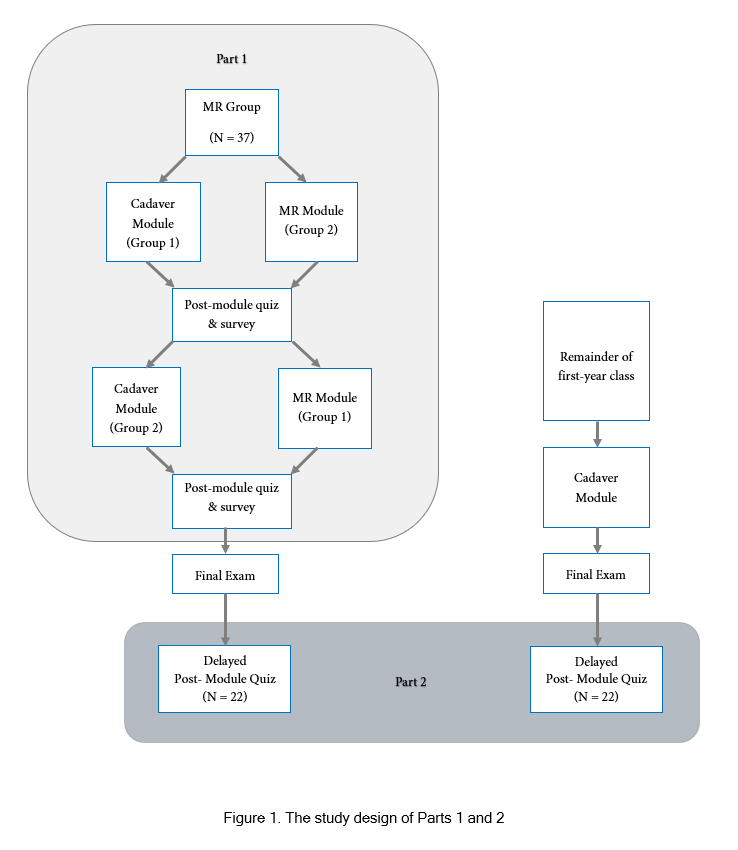
The study design of Parts 1 and 2

Part 2 was a non-randomized controlled trial comparing the performance of study participants and their classmates, who learned the material via cadaveric dissection only, on a seven-item delayed post-module quiz. One hundred and fifty-one first-year medical student volunteers from the same class participated in Part 2 of this study. Of the 151 volunteers in Part 2, twenty-two had participated in Part 1, while the other 129 had no previous experience learning anatomy using MR. The study design of Parts 1 and 2 are described in Figure 1.

Participation in both parts of the study was voluntary, and participants were not compensated in any way. All documents were de-identified and matched using anonymous codes provided by the participants. The protocol for this study was given exempt status by the Institutional Review Board of Case Western Reserve University under 45 Code of Federal Regulations (CFR) part 46.101(b) (1). This protocol includes any investigation conducted in established or commonly accepted educational settings involving normal educational practices, such as research on regular and special education instructional strategies or research on the effectiveness of or the comparison among instructional techniques, curricula, or classroom management methods.

### Intervention

The MR intervention was limited to students who participated in Part 1 of the study. On the day of the MR activity, Part 1 participants were given a 10 to 15-minute introduction to navigating through the views with the MR device. They were also provided with the same dissection manual used by the entire class during the cadaveric breast dissection and access to anatomic atlases. Under the guidance of a staff member, each student donned a MR device. To replicate the small group learning of the dissection lab, a custom networking and display software package was developed for use with the MR device. All students were able to view the same content as their peers and participated in the learning exercise in teams of four, as is the format of the dissection lab. The anatomical material was presented in a manner reminiscent of a PowerPoint presentation through which the models were displayed sequentially. Since the headsets were untethered from a computer, each student was free to walk around the holographic model and view it from any desired perspective. Timing during the module was unrestricted, but students generally completed the lesson within 20 minutes.

All first-year medical students in the CWRU SOM program, including those who participated in the MR module, were required to participate in a cadaveric dissection on the same topic. Teams of four students worked through the dissection manual, attempted to isolate the necessary structures associated with the female breast, and learned the concepts and structures as a group. Those who participated in the MR module completed the cadaveric dissection with the same team of four at the same time as their classmates.

### Data collection

In Part 1, participants completed a comprehension post-module quiz and post-module survey following completion of the MR and cadaver modules. The post-module quizzes consisted of seven questions on the material that had been covered. No feedback was provided to the students after the quizzes in either of the conditions. The post-module surveys consisted of a 5-point Likert scale addressing student perceptions of the module content, understanding of the material presented, ease of learning, ease of performing the task, and ability to work as a team.

Eight weeks after the intervention, between Parts 1 and 2, all class members completed a final exam that required knowledge of all material covered in the breast module. Part 2 occurred eight months after the initial intervention and six months after the final exam. In Part 2, a delayed post-module quiz was offered to the entire class. The delayed post-quiz consisted of seven conceptual questions pertaining to breast anatomy. Knowledge of this material was required for the final exam. Questions were designed to cover topics relating to musculature, lymphatic drainage, blood vessels, fascial layers/connective tissue, and conceptual understanding of milk production and the path of milk flow through the various breast substructures. All questions were reviewed and approved by members of the anatomy department at CWRU SOM.

### Analysis

The Wilcoxon signed-rank test was used to analyze the post-module surveys from Part 1 because the sample was not normally distributed, and differences were measured within-subjects. The Mann-Whitney U test was used to compare post-module quiz results from Part 1 between intervention participants who completed the delayed post-test in Part 2 and those who did not. The Mann-Whitney U test was also used to assess differences in final exam results between students who participated in Part 1 (MR intervention) and the remainder of the class. For Part 2, the Mann-Whitney U test was used to compare delayed post-module quiz results between students who participated in Part 1 (MR intervention) and those who did not. Additionally, ninety-five percent confidence intervals were used to estimate the difference in the proportion of correct responses for each of the delayed post-module quiz questions between Part 1 (MR intervention) participants and Part 2 volunteers who did not receive the MR intervention.

## Results

### Post-Module Survey

The results of the post-module survey comparing participants' reactions after the MR and dissection experiences are detailed in Table 1. In the post-module survey, participants

did not demonstrate a statistically significant difference in their self-assessment of understanding breast anatomy or the ease of performing the designated task (using the MR device or dissecting the breast).  The Wilcoxon signed-rank test showed that the MR group expressed a significantly more positive reaction to using the MR device to study breast anatomy (M = 4.51, N = 37) than to studying breast anatomy via cadaveric dissection (M = 3.95, N = 37), z = -2.97, p < .003. Additionally, the MR group indicated significantly greater ease of learning breast anatomy with MR (M = 1.89, N = 37) than with dissection (M = 3.46, N = 37), z = -3.95, p < .001, and significantly greater ease to work with their team when using the MR device (M = 1.30, N = 37) than when performing dissection (M = 1.73, N = 37), z = -2.13, p < .03.

**Table 1:** Post-Module Survey Responses

Question	Mixed reality	Dissection	p-value
	Mean (SD) (N = 37)	Mean (SD) (N = 37)	
First reaction to breast module	4.51 (0.56)	3.95 (0.91)	< .003^*^
Understanding of Breast Anatomy	2.59 (0.80)	2.72 (0.97)	.62
Ease of learning using modality	1.89 (0.61)	3.46 (0.85)	< .001^*^
Ease of performing task using modality	1.95 (0.78)	2.42 (1.03)	.45
Ability to work as a team using modality	1.30 (0.62)	1.73 (0.93)	.03^*^

^*^p < .05, Wilcoxon Signed-Rank Test

Note:  5-point Likert scale: 1 = Very Negative, 5 = Very Positive for “first reaction to breast module”; all others 1 = Very Easy, 5 = Very Difficult

### Delayed Post-Module Quiz

One hundred fifty-one students completed a delayed post-module quiz eight months after the intervention and six months after the final exam. The Mann-Whitney U test showed no difference in final exam scores between students who participated in the MR modules and the remainder of the class (U = 2,531, p= .23) (Table 2). Before the delayed quiz, no student in either group had any significant clinical experiences with breast anatomy. Twenty-two of the 151 students had been in the MR group, and the remaining 129 students had been in the dissection-only cohort. Twenty-two of the thirty-seven students who participated in the original MR study participated in the delayed post-module quiz. To ensure that those who completed the delayed post-module quiz were a representative sample, the Mann-Whitney U test was used to compare mean scores on the seven-question post-module quiz that was completed immediately following each module. No significant difference was found between the scores of those who did and did not take the delayed post-quiz, indicating similarity (U = 159, p = .87) (Table 3). On the seven-question delayed post-module quiz, the Mann-Whitney U test showed that participants in the MR group scored significantly higher than the control group (U = 928, p < .009) (Table 2).

**Table 2:** Evaluation Scores

Scores	Mixed reality		Control		p-value
	Mean	SD	Mean	SD	
Final Exam	84.9% (N = 43)^*^^*^	6.7	83.5% (N = 134)	8.1	.23
Delayed Post-Quiz	46.1% (N = 22)	21.6	32.8% (N = 129)	16.0	< .009^*^

^*^ p < .05, Mann-Whitney U Test

^**^Six participants withdrew from the study, but could not be identified due to participant anonymity

Ninety-five percent confidence intervals were calculated to estimate the difference in the proportion of correct responses between the MR group and the control group for each of the seven questions (Table 4). The MR group was significantly more likely to provide a correct response for three of the seven questions, whereas there was no significant difference for the other four.

**Table 3:** Mean Post-Module Quiz

Percentage score of students who completed delayed post-test	SD	Percentage score of students who did not complete delayed post-test	SD	p-value
54.5% (N = 22)	17.4	53.3% (N = 15)	18.1	.87

Note: p > .05, Mann-Whitney U Test

**Table 4:** Difference in Proportion of Correct Responses Mixed-Reality Versus Control Group

Question	Mixed reality: Number Correct Responses (%) N = 22	Control: Number Correct Responses (%) N = 129	95% Confidence Interval [CI]
Please name the muscle that lies immediately deep to the breast?	22 (100)	123 (95.3)	[0.0105, 0.0835]
The majority of lymphatic fluid from the breast first drains into which group of lymph nodes?	4 (18.2)	7 (5.4)	[-0.0379, 0.2939]
The artery that supplies the majority of the lateral aspect of the breast originates from which of the following vessels?	8 (36.4)	40 (31.0)	[-0.1623, 0.2703]
The suspensory ligaments of Cooper connect the clavipectoral fascia to which of the following?	10 (45.5)	50 (38.8)	[-0.1574, 0.2914]
Briefly describe the path of milk as it exits the breast?	8 (36.4)	17 (13.2)	[0.0226, 0.4414]
Name the structure that separates the lobes of the breasts?	4 (18.2)	22 (17.1)	[-0.1628, 0.1848]
At which dermatomal level are the nipples located?	15 (68.2)	37 (28.7)	[0.1853, 0.6047]

## Discussion

One of the aims of this study was to evaluate student impressions of MR as a learning modality. Both groups expressed a similar understanding of breast anatomy in the post-module surveys, indicating that the MR module matched the cadaveric module in its educational objectives. Students also found that the ease of using the MR device to perform necessary tasks for learning breast anatomy was similar to traditional dissection. However, the post-module survey suggested that students had a more favorable reaction to the breast module when learning with MR than when performing cadaveric dissection (z = -2.97, p < .003). They also found MR to be an easier modality for learning breast anatomy than cadaveric dissection (z = -3.95, p < .001) and an easier modality to employ for working with their teams (z = -2.13, p < .03).  This is supported by other studies that have found that today's medical students are more engaged and motivated when supplementing their learning with technological modalities.^7^^,^^36^ This notion is in-line with the results of Courteille and colleagues  who found that medical students felt learning with a virtual patient was more stimulating and engaging than learning by way of a lecture format.^3^ The students did not express a difference in understanding breast anatomy when using MR or dissection. This may be because the material was not difficult to understand, even though some of it was difficult to view on the cadaver. The students found neither the MR nor the dissection activities challenging to perform.

Another objective of this study was to determine whether students who supplemented cadaveric dissection with MR would perform better on their final exam and at least as well on a delayed post-test as their peers who only dissected. The results did not support our hypothesis that students who supplemented with MR would perform better on their final exam. Still, they did support our hypothesis that student performance on a delayed post-test would be equivalent or better than that of their peers who only learned via cadaveric dissection. In fact, students in the MR cohort far outperformed their peers on the delayed post-test (U = 928, p < .009), suggesting an MR-mediated improvement in long-term content retention.

To our knowledge, this is the first study to show demonstrable long-term gains in anatomical knowledge when supplementing cadaveric dissection with MR. Results from the delayed post-quiz eight months post-intervention demonstrate that students who supplemented cadaveric dissection with MR performed significantly better than their peers who did not supplement with MR. This is particularly interesting because there was no difference in final exam performance between the two groups two months post-intervention. The final exam results indicate that all students, regardless of whether they supplemented their dissection with MR, had comparable and sufficient understanding of the subject matter earlier in the year. Custers described "retention interval" as a temporal unit during which learned material is not accessed or retrieved and emphasized that retention studies should control for relearning and rehearsal during this interval.^23^ In a subsequent study, Custers emphasized the importance of application or rehearsal in long-term knowledge retention.^24^ None of the first-year medical students had any significant clinical experiences between the initial intervention at the beginning of the year and the delayed post-module quiz at the end of the year, so rehearsal of this knowledge should not have confounded the results.

The entire first-year class was invited to complete the delayed post-module quiz, but it was not mandatory. Some students who participated in the MR modules and some who only completed the cadaveric dissection elected not to take the delayed post-module quiz.  Since no significant difference was found on the post-module quizzes between members of the MR group who took the delayed post-test and those who opted not to, there is no reason to believe that the results reflect a selection bias. It is generally accepted that repeated retrieval of learned material shortens the retention interval and improves retention.^25^^,^^34^^,^^35^ In the present study, the MR group had one additional opportunity to retrieve the material they learned prior to their examination. Since no difference was found between the MR cohort and their peers on the final exam, it stands to reason that this additional exposure did not influence the long-term retention of the material.

While this study does not provide information on a change in knowledge, it suggests that supplementing cadaveric dissection with MR may increase the capacity to retain acquired knowledge over a greater retention interval. Jurjus suggested that students and clinicians alike recognize the value of the vertical integration of anatomical concepts into curricula.^22^ Custers recommended variable practice as well as rehearsal and restudy of basic science knowledge at regular intervals to increase retention.^23^ The convenience and flexibility of MR make it an ideal medium for senior medical students, residents, and even practicing physicians to review anatomical material expeditiously. The goal for medical educators is to prepare students for their clinical clerkships and future careers as physicians, not merely to pass exams. A pedagogic method that increases the long-term retention of material serves this goal and merits further exploration.

One limitation of this study is that the novelty of the MR may have impacted participant satisfaction with the modality. Prior research has indicated that greater perceived satisfaction and effectiveness of 3D visual techniques over 2D anatomical images may be due to the novelty of the technology.^37^ Also, the subjective data in this study was reported using self-reporting questionnaires, which can include subjective biases. The small sample size of the intervention and the small number of quiz questions may also introduce bias. Due to the de-identification procedure used in this study, some study participants could not remember their personal codes at the time of the delayed post-test eight months after the intervention, so the researchers were unable to compare individual scores on the post-test and delayed post-test of the MR group. This further limited our sample size. These results also reflect a single institution's experience using a single type of MR device and may not be generalized to all forms of MR learning. Despite these limitations, the authors believe that these data provide a unique starting point for a novel educational intervention. Repeating these and similar experiments with a more robust cohort of student volunteers who are already exposed to MR could lessen the impact of novelty and provide further clarity on the effectiveness of MR supplementation in anatomy education.

Additionally, given the evidence that medical students perceive the learning of different anatomical regions as having differing degrees of difficulty,^41^ it is possible that MR is particularly effective for the study of female breast anatomy, which includes structures that cannot easily be viewed macroscopically. Swanson et al. has determined that knowledge concerning specific organ systems is better retained than knowledge of a more general nature that cannot be categorized into a single system,^38^ and investigation of a focused educational topic has been identified as a limitation by other authors.^39^^,^^40^ As such, future investigation would be necessary to assess the effectiveness of our combined educational approach in teaching a broader range of anatomical topics.

These results demonstrate promise for MR as a supplement to cadaveric dissection; however, further research is needed to understand the factors contributing to the improved scores. For instance, it is currently unclear whether long-term retention would have improved if cadaveric dissection was supplemented by another technological modality or if the results were due to the added value provided by the MR assets. It would also be of great value to assess retention prior to third-year clerkships when anatomical knowledge will need to be applied in clinical settings.

## Conclusions

The purpose of the present study was to gather student feedback about their experience using MR as a supplement to cadaveric dissection and to compare long-term retention of information about female breast anatomy between students who supplemented their initial learning with MR and their classmates who did not. The post-module surveys suggest that medical students may prefer MR over cadaveric dissection and found it to be an easier modality for learning breast anatomy. The results also suggest that MR facilitates teamwork more readily than cadaveric dissection. This study also suggests that supplementing cadaveric dissection with MR can improve long-term retention for at least one anatomical topic, the female breast. Finally, MR may provide a quick and effective tool in conjunction with cadaveric dissection to improve the retention of anatomical concepts over long periods of time. This feedback is very encouraging when considering the integration of MR into a curriculum. If supplementing cadaveric dissection with MR can aid medical students in retaining more anatomical knowledge, the field of medical education will benefit from this approach by producing better prepared students for their clinical clerkships and future careers. It is recommended that studies of this sort be conducted with a more robust sample size across additional anatomical regions to determine the generalizability of these findings.

### Conflict of Interest

The authors declare that they have no conflict of interest.
